# Keyhole surgery of CTS using novel tool MiniSure

**DOI:** 10.1186/1753-6561-9-S3-A79

**Published:** 2015-05-19

**Authors:** Sunton Wongsiri

**Affiliations:** 1 Department of Orthopaedic Surgery, Prince of Songkhla University, Songkhla, 90110, Thailand

## Introduction

The standard open technique for carpal tunnel surgery has wound problems and complications significantly more than minimally invasive surgery using the keyhole Wongsiri technique with MiniSURE, and in particular, the open technique surgery requires a longer time for return to work. CTR surgery with endoscopic devices improves the results with fewer wound problems when compared to the commonly used open technique; however, nerve complications and injury are more prevalent with endoscopic surgery than with the open technique. The keyhole Wongsiri technique produces good results with new medical devices such as the MiniSURE View, for improved vision and line-of-sight, and the MiniSURE Cut for improved and complete cutting via the supraretinacular technique that may reduce the nerve problems associated with endoscopic tooling in the carpal tunnel.

## Background and aims

To evaluate the results of the operation and postoperative outcomes of the Wongsiri technique with a MiniSURE kit.

Material and Methods: 20 patients underwent carpal tunnel release using the Wongsiritechnique and a MiniSURE kit with a five-step surgery: MIS starts when the surgeon makes a 1.5-1.8 cm incision, creates a working space, inserts the visual tube ofMiniSURE View, inserts the freer, then cuts the transverse carpal ligament by using theMiniSURE Cut.

## Results and conclusions

All 20 successes of the keyhole Wongsiri technique and MiniSURE kit surgery occurred within 6.8 minutes operative time and a 12 mm. wound size. A single outlier, in one case (6.7%), the patient experienced pillar pain which abated within one month. Patients can return to work in 7.3 days. The Wongsiri technique with theMiniSURE kit demonstrated good outcomes similar to the endoscope. By contrast with the endoscopic surgery, the Wongsiri technique with the MiniSURE kit reduced pre-op, operating and post-op time, many resources, and significant costs and resulted in no nerve problems or complications.

